# Analysis of the response of the cell membrane of *Saccharomyces cerevisiae* during the detoxification of common lignocellulosic inhibitors

**DOI:** 10.1038/s41598-021-86135-z

**Published:** 2021-03-25

**Authors:** Pau Cabaneros López, Chuantao Peng, Nils Arneborg, Helena Junicke, Krist V. Gernaey

**Affiliations:** 1 Process and Systems Engineering Center (PROSYS), Department of Chemical and Biochemical Engineering, Technical University of Denmark (DTU), Building 228A, 2800 Lyngby, Denmark; 2 Department of Food Science, University of Copenhagen (KU), Rolighedsvej 26, 1958 Frederiksberg C, Denmark

**Keywords:** Environmental biotechnology, Industrial microbiology, Flow cytometry, Cell biology

## Abstract

Gaining an in-depth understanding of the response of *Saccharomyces cerevisiae* to the different inhibitors generated during the pretreatment of lignocellulosic material is driving the development of new strains with higher inhibitor tolerances. The objective of this study is to assess, using flow cytometry, how three common inhibitors (vanillin, furfural, and acetic acid) affect the membrane potential, the membrane permeability and the concentration of reactive oxygen species (ROS) during the different fermentations. The membrane potential decreased during the detoxification phase and reflected on the different mechanisms of the toxicity of the inhibitors. While vanillin and furfural caused a metabolic inhibition and a gradual depolarization, acetic acid toxicity was related to fast acidification of the cytosol, causing an immediate depolarization. In the absence of acetic acid, ethanol increased membrane permeability, indicating a possible acquired tolerance to ethanol due to an adaptive response to acetic acid. The intracellular ROS concentration also increased in the presence of the inhibitors, indicating oxidative stress. Measuring these features with flow cytometry allows a real-time assessment of the stress of a cell culture, which can be used in the development of new yeast strains and to design new propagation strategies to pre-adapt the cell cultures to the inhibitors.

## Introduction

The production of ethanol from lignocellulosic material is largely considered as a potential source of renewable fuel from agricultural or forestry waste. During the last three decades, extensive research has been carried out in the field to optimize the different stages of the conversion of lignocellulosic material into ethanol. Despite the successful demonstration at lab and pilot scales, cellulosic ethanol did not become a reality at an industrial scale yet^1^. An important reason is that many compounds, generated during the pretreatment of the lignocellulosic biomass, have inhibitory effects on the enzymatic hydrolysis and the fermentation steps, where genetically modified strains of *Saccharomyces cerevisiae* are typically used to convert the C6 and C5 sugars into ethanol. These inhibitory compounds are derived from the partial degradation of the cellulose, hemicellulose, and lignin present in the lignocellulosic material, and can be classified into weak acids (e.g. acetic acid), furan derivates (e.g. furfural or 5-hydroxymethylfurfural (5-HMF)) and phenolic compounds (e.g. vanillin or 4-hydroxybenzoic acid)^2,3^. The inhibitory effects of the different compounds are complex and not fully understood^4,5^. Also, interactions between the different inhibitors further aggravate the overall inhibitory effects^6^. Ethanol has also been identified as an inhibitor of yeast growth, especially at high titers (> 10% v/v)^7,8^. Understanding the regulatory mechanisms and effects of the different inhibitors on cell physiology is key to developing yeast strains with high tolerance towards growth inhibitors, and to increase the performance of cellulosic ethanol processes.

Upon contact with the inhibitors (e.g. vanillin, furfural or acetic acid) *S. cerevisiae* can assimilate them (e.g. vanillin or acetic acid^5,9^) or can convert them into less inhibitory compounds (e.g. furfural is oxidized to furoic acid or reduced to furfuryl alcohol under aerobic or anaerobic conditions, respectively^5^). In addition to detoxify the fermentation media, *S. cerevisiae* also undergoes several physiological and metabolic changes as a consequence and as a response to the inhibitors^6,10,11^. Among these changes, rearrangements of the cell membrane composition have been previously described as a response to ethanol^12,13^, acetic acid^14,15^ phenolic compounds^16,17^ and furan derivates^18^. The effect of ethanol on the membrane of *S. cerevisiae* has been widely studied in the last 30 years^8^. Increased permeabilization of the yeast membrane, when exposed to ethanol, has been reported both experimentally^7,8,19–24^ and computationally using molecular dynamics^25^. Also, changes in the membrane composition of *S. cerevisiae* have been reported under ethanol stress. Chi et al.^12^ compared the lipidic composition of the membranes of two strains of *S. cerevisiae* growing at 18% v/v of ethanol. They showed that the strain exhibiting a higher ethanol tolerance contained a higher ergosterol concentration (2 mg/g of cell dry weight (CDW) higher). Moreover, Arneborg et al.^13^ studied the adaptative response *of S. cerevisiae* growing at 1% v/v and 6.4% v/v of ethanol, and showed that the higher concentration of ethanol induced a higher content of ergosterol (from 1.65 to 2.15 mg/g of CDW). A higher ergosterol concentration is associated with a reduced membrane fluidity and a higher ethanol tolerance (at 10% v/v of ethanol)^20^. Similarly to ethanol, acetic acid also influences the composition of the cell membrane promoting an adaptive response to increase its tolerance towards acetic acid^26^. Lindberg et al.^15^ studied the lipidomic profile of *S. cerevisiae* in response to acetic acid (4.0 g/L) and compared it with that of *Zygosaccharomyces bailii*, an acetic acid tolerant yeast strain able to grow at concentrations of 10.5 g/L of acetic acid. In the absence of acetic acid, *Z. bailii*, showed a higher concentration of complex sphingolipids than *S. cerevisiae*. However, in the presence of acetic acid, the concentration of complex sphingolipids increased in both, *Z. bailii* and *S. cerevisiae*, suggesting their contribution to increase the resistance to acetic acid. This was further supported by simulating the bilayer membrane of *Z. bailii* using molecular dynamics, which showed that a higher content of complex sphingolipids increased the thickness and the density of the cell wall^15^. Endo et al.^16,17^ suggested that ergosterol was also related to vanillin tolerance, after finding that vanillin-resistant *S. cerevisiae* had higher concentrations of cytosolic ergosterol and overexpressed genes involved in the ergosterol biosynthesis. For furfural, unlike with the previous compounds, there is no evidence that it alters the membrane lipid composition^27^. However, Wu et al.^18^ have recently shown the importance of the membrane multidrug resistance (MDR) complexes in the development of tolerance for various furan derivates.

The previous studies demonstrate the importance of the cell membrane in the development of tolerance to inhibitors commonly found in lignocellulosic hydrolysate. This has motivated the development of improved strains by engineering the cell membranes^28–30^. In this context, improving the understanding of cell membrane properties is key to developing novel strains. Narayanan et al.^31^ have previously used flow cytometry to provide valuable insights on the effect of vanillin (0.16–1.84 g/L), furfural (0.16–1.84 g/L) and acetic acid (1.00–7.70 g/L) on the internal pH, the cell viability and the oxidative stress of *S. cerevisiae*, and Freitas et al.^32,33^ also used flow cytometry to study the effects of acetic (0–15 g/L) and formic acid (0–20 g/L) on the membrane potential, viability and oxidative stress of *Saccharomyces carlsbergensis*. However, none of the previous studies examined the effects of different combinations of lignocellulosic inhibitors on the cell membrane of *S. cerevisiae*.

The objective of the present study is to systematically examine the dynamic changes in the membrane integrity of an industrial strain of *S. cerevisiae* (Ethanol Red^®^) in response to the presence of different combinations of lignocellulosic inhibitors (vanillin, furfural and acetic acid) throughout fermentations. Flow cytometry and fluorescent molecular probes were used to assess changes in the membrane potential and the membrane porosity. The membrane potential was measured using DiOC_6_(3), a fluorescent dye capable of penetrating membranes of polarized cells^34^. The membrane porosity was studied using the fluorescent stain propidium iodide (PI), which penetrates and stains only cells with perforated membranes^22,35^. In addition, the concentration of intracellular reactive oxygen species (ROS) was also monitored as an indicator of the oxidative stress using the stain DHR123^36,37^, a non-fluorescent stain that penetrates the cells and becomes fluorescent when it reacts with H_2_O_2_ in the presence of endogenous peroxidases^38^.

## Results

### Fermentation profiles under different inhibitors

**Figure 1:**
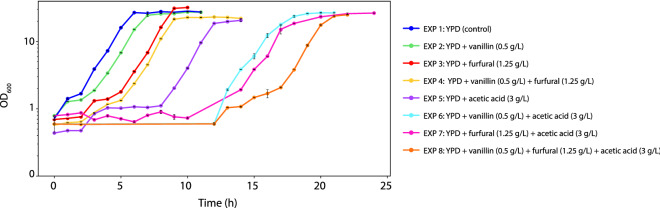
Growth profiles of *Saccharomyces cerevisiae* in the different experiments. The values plotted are the average of two replicates, and the error bars indicate the difference between the two replicates. This figure was produced using Python^70^.

**Figure 2:**
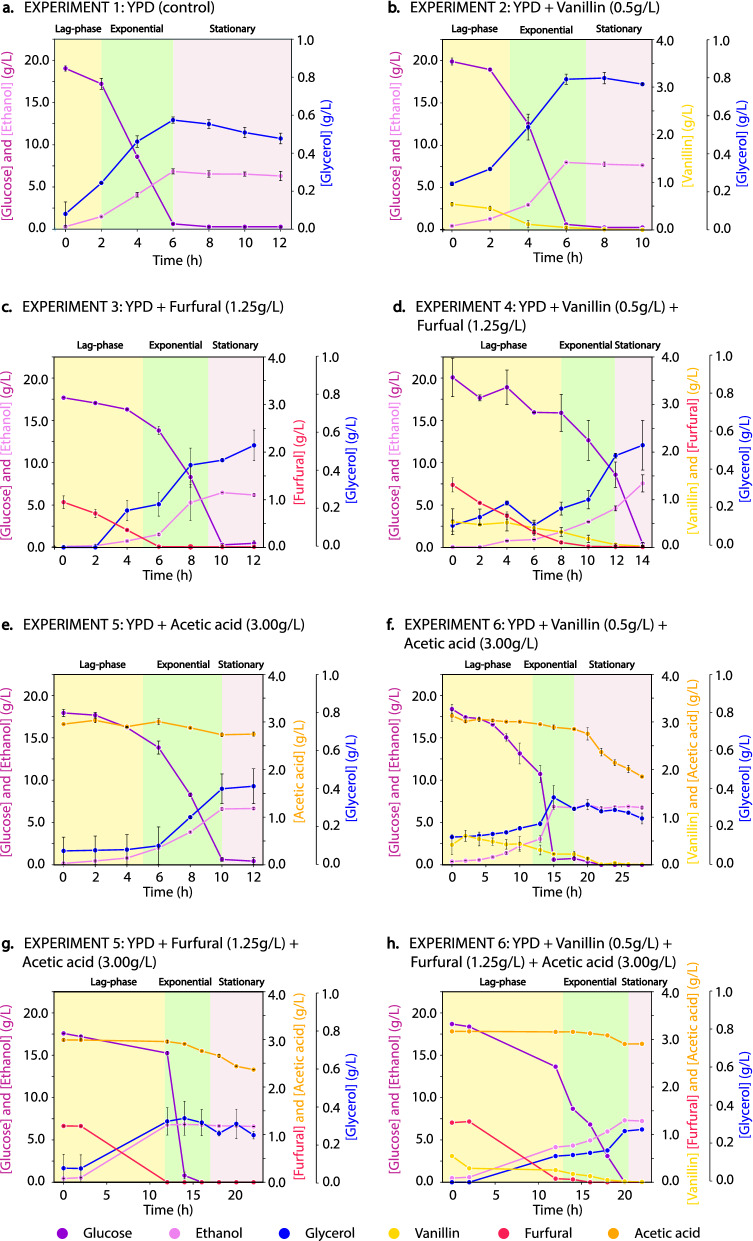
Profile of the extracellular metabolites through experiments 1–8. The plots show substrate (glucose), product (ethanol and glycerol) and inhibitors (vanillin, furfural and acetic acid) concentrations measured during each fermentation. (**a**) YPD control, (**b**) addition of vanillin (0.5 g/L), (**c**) addition of furfural (1.25 g/L), (**d**) addition of vanillin (0.5 g/L) and furfural (1.25 g/L), (**e**) addition of acetic acid (3 g/L), (**f**) addition of vanillin (0.5 g/L) and acetic acid (3 g/L), (**g**) addition of furfural (1.25 g/L) and acetic acid (3 g/L), (**h**) addition of vanillin (0.5 g/L) furfural (1.25 g/L) and acetic acid (3 g/L). For fermentations 1–7, the plotted values are the average of two replicates and the error bars indicate the difference between the two replicates. Note that for fermentation 8, only one replicate was measured. This figure was produced using Python^70^.

The fermentation profiles for the experiments 1–8 are shown in Figs. 1 and 2, while the specific growth rate, the lag phase, the ethanol yield and the maximum glycerol concentration are shown in Table 1. Under the experimental conditions, increasing the number of inhibitors in the fermentation media resulted in an increased length of the lag phase. Comparing the lag phase of the control experiment with the experiments containing each inhibitor alone (Fig. 1), the length of the lag phase increased by 1, 3 and 3 h respectively, when vanillin, furfural or acetic acid (HAc) were present in the media. The extended lag phase corresponded to a detoxification phase in which *S. cerevisiae* adapts and responds to the inhibitors. Different detoxification profiles can be seen by comparing Fig. 2a–c,e. The concentration of vanillin and furfural decreased during the lag phase because they were assimilated (vanillin^5,9^) or detoxified into less inhibitory compounds (furfural is often converted into furoic acid or furfuryl alcohol under aerobic and anaerobic conditions, respectively^5^). Moreover, while detoxification of vanillin occurred simultaneously to cell growth (Figs. 1 and 2b), detoxification of furfural happened prior to cell growth (Figs. 1 and 2c). The concentration of acetic acid neither changed considerably during the lag phase nor during the growth phase (Fig. 2e). The pH of the fermentation media was measured at the beginning of the fermentation and during the growth phase using pH test strips. The initial pH of the fermentations containing acetic acid was 3.5 ± 0.5 meaning that 90% of the acetic acid (2.7 g/L) was found in the protonated form (the pKa of acetic acid is 4.76). The initial pH of the remaining fermentations was 5.5 ± 0.5. After the fermentations, the pH was 4.5 ± 0.5 in all the fermentations. When *S. cerevisiae* was cultivated with two inhibitors simultaneously (Figs. 1 and 2d,f,g), the length of the lag phase increased by + 6, + 8 and + 8 h for the pairs vanillin-furfural, vanillin-acetic acid and furfural-acetic acid, respectively. A similar profile was obtained when all the inhibitors were present simultaneously (Figs. 1 and 2h), in which the lag phase was + 10 h longer than observed for the control experiment (Figs. 1 and 2a).

**Table 1:** Specific growth rate (µ), lag-phase, ethanol yield (Y_ethanol/glucose_) and maximum glycerol concentration in experiments 1–8.

Experiment Nr	Vanillin (g/L)	Furfural (g/L)	Acetic acid (g/L)	Lag phase (h)	µ (/h)	Y_ethanol/glucose_ (g/g)	Max glycerol (g/L)
1	0.00	0.00	0.00	2	0.70 ± 0.001	0.36 ± 0.011	0.57 ± 0.018
2	0.50	0.00	0.00	3	0.66 ± 0.009	0.40 ± 0.006	0.66 ± 0.157
3	0.00	1.25	0.00	5	0.72 ± 0.001	0.36 ± 0.001	0.54 ± 0.081
4	0.50	1.25	0.00	8	0.73 ± 0.001	0.38 ± 0.009	0.54 ± 0.131
5	0.00	0.00	3.00	5	0.72 ± 0.002	0.37 ± 0.002	0.41 ± 0.093
6	0.50	0.00	3.00	10	0.59 ± 0.012	0.39 ± 0.001	0.35 ± 0.027
7	0.00	1.25	3.00	10	0.56 ± 0.001	0.39 ± 0.047	0.34 ± 0.078
8	0.50	1.25	3.00	12	0.64 ± 0.032	0.39 ± n.d	0.27 ± n.d

### Effect of the different inhibitors on the cell physiology

#### Reproducibility of the flow cytometry data

**Figure 3:**
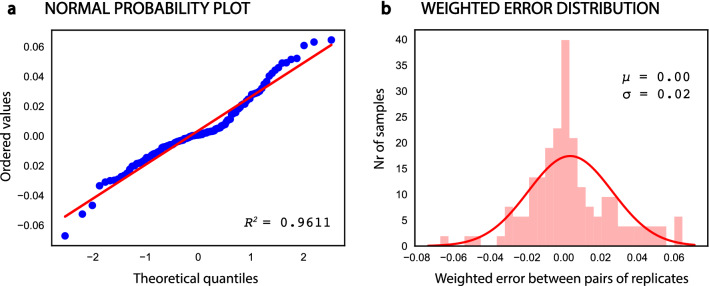
Analysis of the error between replicates of the flow cytometry data. (**a**) normal probability plot of the experimental data. The red line represents an ideal normal distribution. The high R^2^ coefficient of the experimental data with the red line indicates that the weighted errors are nearly normally distributed. (**b**) Weighted error distribution, where µ is the mean value of the distribution and σ is the standard deviation. The red line shows the normal distribution fitted to the experimental weighted errors. This figure was produced using Python^70^.

The weighted error (equation (1)) between each pair of replicated cytograms was used to assess the reproducibility of the measurements taken with the flow cytometer. In the absence of systematic errors, the weighted error of all sample pairs should follow a normal distribution with 0 mean and ${\sigma }_{error}$ standard deviation $\left(\delta \left(error\right)\sim N(0,{\sigma }_{error})\right)$. Moreover, deviations between pairs of replicates would result in large standard deviations (${\sigma }_{error}$). The normality of the distribution of weighted errors was assessed using a normal probability plot (a graphical method to assess if a set of data is approximately normally distributed^39^), which showed no significant deviations from normality (Fig. 3a). Therefore, a normal density function was fitted to the error distribution (Fig. 3b), resulting in a distribution with 0 mean and standard deviation of 0.02 ($\delta (error)\sim N(\mathrm{0,0.02})$). This indicates that 95% of the measurements (2 standard deviations) had a weighted error below 4% and that no systematic error was introduced during the experiment, demonstrates a good reproducibility of the experimental method.

#### Forward and side scatter

**Figure 4:**
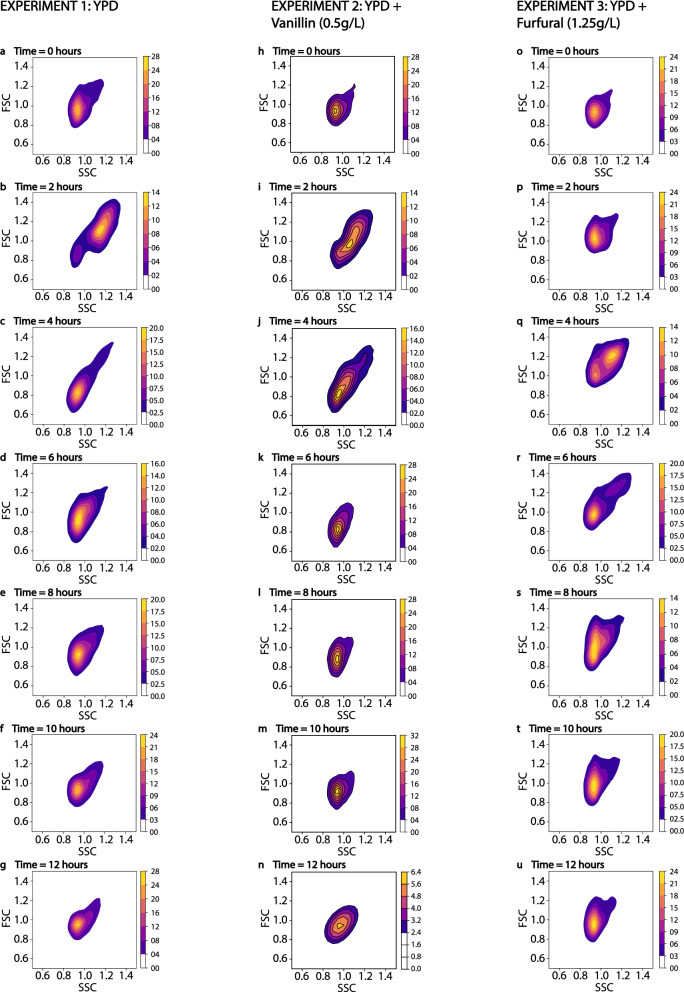
2-D density plots showing the evolution of the forward and side scatter. The density plots correspond to experiment 1 (**a**–**g**), experiment 2 (**h**–**n**) and experiment 3 (**o**–**q**). Notice that the FSC and SSC are scaled by the mean FSC and SSC of experiment 1 at time 0 h. Hence, all measured changes in the population of cells are given in relation to that state. The colour gradient shows the density of the distribution in the populations, with yellow corresponding to the highest density, and blue corresponding to the lowest one. This figure was produced using Python^70^.

The forward and side scatter (FSC and SSC respectively) evolved following a similar profile throughout all the fermentations. This profile is shown for experiment 1 (Fig. 4a–g) and for experiment 2 (Fig. 4h–n) and for experiment 3 (Fig. 4o–u). The profiles for all the other fermentations are provided in the Supplementary Material (Figs. SM1, SM2 and SM3). The lag phases were characterized by a single uniform cell population with a small ‘tail’ with larger FSC and SSC (Fig. 4a,h–I,o–q). This population remained stable during the lag phases of all the fermentations. At the beginning of the growth phase, a second cell population with larger FSC and SSC developed (Fig. 4b,q). In some fermentations, such as in experiment 2 (Fig. 4h–n), the distinction between the two populations was not so obvious (Fig. 4i,j). The fraction of this second population decreased during the growth phase (Fig. 4c,k,r) until only a single cell population remained at the late-growth and early-stationary phases (Fig. 4d,l,s). This new cell population resembled the cell population of the lag phase, but it was more spread (i.e. it had a similar mean for the FSC and the SSC compared to the lag phase but higher standard deviation). During the stationary phase, the cell population contracted considerably becoming more homogeneous (Fig. 4g,l–n,t,u). At the end of the fermentations, the cell population reached a size similar to that observed in the lag phase.

#### Fluorescence

##### Changes in the membrane potential

**Figure 5:**
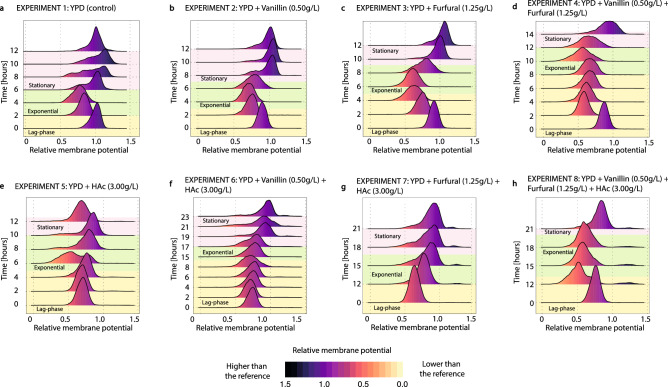
Evolution of the membrane potential of the cell culture during the experiments 1–8. The membrane potential (measured using DiOC_6_(3)) is scaled by the mean of the membrane potential in experiment 1 at time 0 h (**a**). The colour code indicates how far a population is from the reference point (i.e. orange and yellow indicate a membrane potential lower than that of the reference population, while black and dark purple indicate a higher membrane potential than in the reference population). This figure was produced using R^71^. (**a**) YPD control, (**b**) addition of vanillin (0.5 g/L), (**c**) addition of furfural (1.25 g/L), (**d**) addition of vanillin (0.5 g/L) and furfural (1.25 g/L), (**e**) addition of acetic acid (3 g/L), (**f**) addition of vanillin (0.5 g/L) and acetic acid (3 g/L), (**g**) addition of furfural (1.25 g/L) and acetic acid (3 g/L), (**h**) addition of vanillin (0.5 g/L) furfural (1.25 g/L) and acetic acid (3 g/L).

The changes in the membrane potential during the eight fermentations are shown in Fig. 5. The control experiment (Fig. 5a) shows that the membrane potential is reduced during the exponential growth, and it is restored during the early stationary phase. This is well illustrated at 6, 8 and 10 h, where the depolarized population gradually recovered its membrane potential, showing a wide distribution of the cell population. The presence of vanillin or furfural caused a gradual depolarization of the membrane during the lag phase, and a progressive repolarization after the growth phase (Fig. 5b,c). Furfural had a more severe effect on the membrane potential causing larger membrane depolarization. This situation was further aggravated when vanillin and furfural were present together (Fig. 5d), where *S. cerevisiae* had a lowered membrane potential during the long lag and growth phases and recovered it at the beginning of the stationary phase. The Fig. 5e–h show the changes in the membrane potential for the experiments where acetic acid was present (experiments 5–8). Unlike vanillin and furfural, acetic acid caused an immediate depolarization of the cell membrane, which remained constant during the lag and growth phases. It was not possible to monitor the entire lag phase for the experiments 7 and 8 (Fig. 5g,h) and only the initial point, and the growth and early stationary phases were studied. The results showed the largest reduction in the membrane potential in the experiment containing all the three inhibitors (Fig. 5h). Moreover, a second population of hyperpolarized cells was detected during the growth phase in the experiments 7 and 8 (Fig. 5g,h).

##### Changes in the membrane permeability

**Figure 6:**
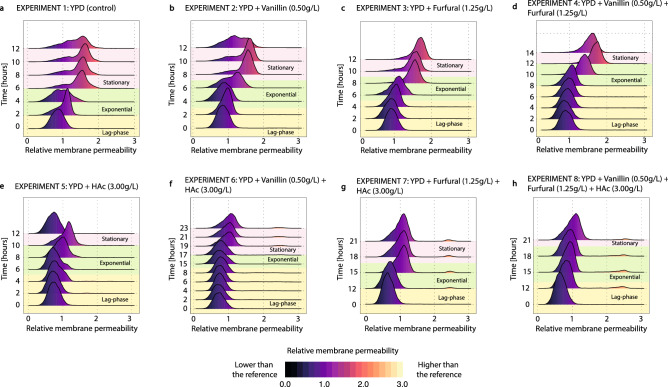
Evolution of the membrane permeability of the cell culture during the experiments 1–8. The membrane permeability (measured using PI) is scaled by the mean of the membrane permeability in experiment 1 at time 0 h (**a**). The colour code indicates how far a population is from the reference point (i.e. orange and yellow indicate a higher membrane permeability than that of the reference population, while black and dark purple indicate a lower membrane permeability than in the reference population). This figure was produced using R^71^. (**a**) YPD control, (**b**) addition of vanillin (0.5 g/L), (**c**) addition of furfural (1.25 g/L), (**d**) addition of vanillin (0.5 g/L) and furfural (1.25 g/L), (**e**) addition of acetic acid (3 g/L), (**f**) addition of vanillin (0.5 g/L) and acetic acid (3 g/L), (**g**) addition of furfural (1.25 g/L) and acetic acid (3 g/L), (**h**) addition of vanillin (0.5 g/L) furfural (1.25 g/L) and acetic acid (3 g/L).

The membrane porosity was assessed using propidium iodide. The changes in the membrane permeability for the batch experiments 1–8 are shown in Fig. 6. In the experiments 1–4 (all without acetic acid), the membrane permeability did not change considerably during the lag and growth phases. However, the membranes became increasingly more permeable (~ 1.75 times higher signal) during the stationary phase (Fig. 6a–d). In the fermentations 5–8 (all containing acetic acid), the membrane permeability did not significantly change during the lag, growth or stationary phases. However, a second small cell population with very high membrane permeability (~ 2.5 times higher signal) appeared during the growth and stationary phases in the fermentations 6–8 (Fig. 6f–h). This second population with higher PI fluorescence is typically associated with non-viable cells^31–33^.

##### Changes in the ROS concentration

**Figure 7:**
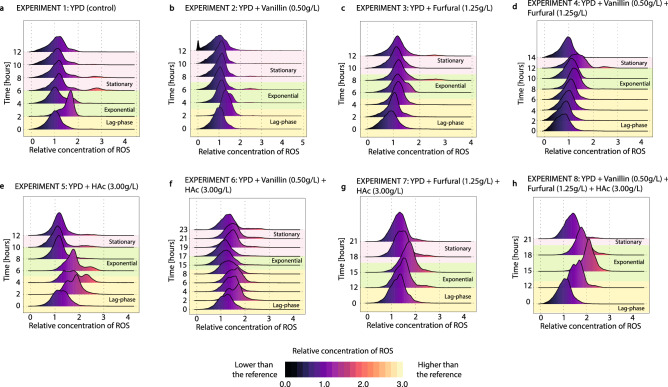
Evolution of the ROS concentration inside the cells during the experiments 1–8. The ROS concentration (measured using DHR123) is scaled by the mean of the ROS concentration in experiment 1 at time 0 h (**a**). The colour code indicates how far a population is from the reference point (i.e. orange and yellow indicate a higher ROS concentration than that of the reference population, while black and dark purple indicate a lower ROS concentration than in the reference population). This figure was produced using R^71^. (**a**) YPD control, (**b**) addition of vanillin (0.5 g/L), (**c**) addition of furfural (1.25 g/L), (**d**) addition of vanillin (0.5 g/L) and furfural (1.25 g/L), (**e**) addition of acetic acid (3 g/L), (**f**) addition of vanillin (0.5 g/L) and acetic acid (3 g/L), (**g**) addition of furfural (1.25 g/L) and acetic acid (3 g/L), (**h**) addition of vanillin (0.5 g/L) furfural (1.25 g/L) and acetic acid (3 g/L).

The changes in the concentration of ROS compounds in each experiment are shown in Fig. 7a–h. Compared to the membrane potential and the membrane permeability, the intracellular ROS concentration showed populations with wider distribution (i.e. with larger standard deviations), indicating higher heterogeneity within the same population. The control experiment showed an accumulation of ROS during the growth phase, followed by a decline during the late-growth and early stationary phases (see Fig. 7a at 0, 2, 4 and 6 h). A gradual accumulation of ROS during the extended lag phases and the growth phase was also found in all the experiments containing inhibitors (Fig. 7b–h). Cell cultures grown in the presence of acetic acid showed a larger accumulation of ROS than those containing furfural or vanillin.

## Discussion

In the present study, the evolution of targeted cell membrane features (i.e. membrane potential or membrane permeability) was monitored in the course of eight batch fermentations to systematically study the effects of the inhibitors vanillin, furfural, and acetic acid on the physiology of *S. cerevisiae*. Additionally, the intracellular ROS concentration, ethanol production, the accumulation of glycerol, and the fermentation profiles were measured to provide a broader understanding of how *S. cerevisiae* responds to the aforementioned inhibitors.

Vanillin, furfural, and acetic acid correlated with a considerable increase in the duration of the lag phase but only caused a small reduction of the specific growth rate, indicating the capability of the cell culture to recover after detoxification (Fig. 1 and Table 1). Similar results have been shown previously for vanillin (0–2.3 g/L)^9,16^ for furfural (0–5 g/L)^6,40^ and acetic acid (0–10 g/L)^6^. However, the effect of the inhibitors on the lag phase and the specific growth rate is highly dependent on their concentration, meaning that higher titers of an inhibitor would result in stronger inhibition effects^6^. The specific inhibitor concentration depends on the feedstock and the pretreatment method^41^. In this study, the concentration of inhibitors was chosen to reflect what is typically observed in wheat straw hydrolysates^42^. The presence of vanillin resulted in a lag phase that was 1 h longer than the control experiment, and furfural and acetic acid caused a lag phase that was 3 h longer than the control experiment (Fig. 1 and Table 1), suggesting that, at these concentrations, vanillin exerts a lower inhibitory effect than furfural or acetic acid. Previous studies have found that vanillin (at 1.4 g/L) reduces the ethanol yield^9^, that furfural increases the ethanol yield when present at low concentrations (0.6 g/L) and decreases it when present at concentrations higher than 2 g/L^6^ and that acetic acid (5 g/L) can increase the ethanol yield^6^. Moreover, a considerable reduction in the ethanol yield was also found as a consequence of the synergic effects of furfural and acetic acid^6^. In the present study the inhibitors did not reducuce the ethanol yield, probably due to the different concentrations used herein (Table 1). Palmqvist et al.^5,41^, have shown that the specific growth rate is more affected by the inhibitors than the production of ethanol, which is consistent with the results found here (Table 1). The maximum glycerol concentration was considerably reduced when furfural or acetic acid were present in the fermentation media (Table 1). This reduction in glycerol production by *S. cerevisiae* in the presence of furfural has been previously reported in the literature^40,43,44^. In normal conditions, *S. cerevisiae* produces glycerol to re-oxidize the surplus of NADH generated during the cell growth and to maintain the redox balance inside the cell^40,43^. However, in the presence of furfural, the excess of NADH is re-oxidized during the detoxification of furfural into the weaker inhibitor furfuryl alcohol^40^, resulting in a decreased glycerol production^40,43,44^. Acetic acid is also known to affect glycerol production as a consequence of the inhibition of the glycolytic pathway^45^.

The forward and side scatter in the flow cytograms of *S. cerevisiae* populations provide physical information about the size and internal complexity (e.g. granularity) of the cell cultures, respectively^46^. The lag phases were characterized by a single cell population skewed towards a higher forward and side scatter (Fig. 4a,h,o). The inhibitors reduced the cell population making it more homogeneous, and reduced the cell size slightly (Fig. 4h–u). The effect of some inhibitors on the size distribution of *S. cerevisiae* was previously studied by Tibayrenc et al.^47^, who found that adding 5 g/L of furfural or acetic acid to an exponentially growing cell culture of *S. cerevisiae* caused a slight reduction of the mean cell diameter and a considerable loss of cell viability^47^. The different responses from both studies are possibly due to the increased toxic effects of the inhibitors when inoculated to exponentially growing cell cultures with a considerable concentration of ethanol (5% v/v) already present in the media^47^. During the growth phase, a second population with a larger size appeared in the experiments 1, 3, 5 and 6 (Fig. 4a–g,o–u for experiments 1 and 3 respectively). Probably, this population corresponded to budding yeast^48^. In experiments 2, 4 and 7 the two populations were not clearly segregated (Fig. 4h–n for experiment 2) and appeared as a single population with a larger size (Fig. 4i,j). The different inhibitors did not significantly affect the properties of this second population, most probably because the inhibitors were detoxified before the cell growth started. As *S. cerevisiae* consumed the glucose, the fraction of budding yeast decreased until it became one single population at the end of the exponential phase. This cell population had a similar distribution as in the lag phase of the experiment without inhibitors, but spanned over a larger range of cell sizes. During the stationary phase the cell population gradually became more homogeneous.

The ability to keep the membrane potential constant is fundamental to maintain the internal homeostasis in *S. cerevisiae*^49^*.* For this reason, many active and passive ion transporters are involved in keeping the membrane potential stable^49,50^. However, under certain stress conditions, *S. cerevisiae* becomes unable to maintain the potential through the membrane, which becomes less polarized. This is why assessing the membrane potential is commonly used as an indirect indicator of cellular stress^32,33^. The present study shows that the membrane potential is a very dynamic property and that it does not only change due to cellular stress, but it also changes during the different phases of the fermentation in non-stressed cell cultures (Fig. 5). During the growth phase in the control experiment, the membrane potential decreases as a consequence of the dynamic metabolic changes associated to cell growth (Fig. 5a). However, the inhibitors aggravate the depolarization of the cell membrane. Vanillin and furfural caused a gradual depolarization during the extended lag phase (Fig. 5b,c). The inhibition mechanisms of vanillin and furfural are complex and not completely understood^5,51,52^, but both compounds are known to inhibit the glycolysis^40^. Vanillin induces granulation of the mRNA content in the cell and promotes mitochondrial fragmentation, often resulting in severe metabolic malfunctions^17,53–55^. Furfural induces stress responses and affects the internal structures of the cells including alterations in the mitochondrial and vacuole membranes, in the actin cytoskeleton or in the nuclear chromatin^56,57^. Cell growth can only occur after the inhibitors have been assimilated or detoxified and transformed into less inhibitory compounds^5,17,40,43,44,58^. When the concentration of the inhibitors is high enough, the detoxification stage is lengthened, compromising the integrity of the cell culture. Furfural caused a longer and more intense depolarization of the cell membrane than vanillin, suggesting that, at the concentrations used throughout this work, furfural had a stronger inhibitory effect than vanillin (Fig. 5b,c). This is further supported by the longer lag phase required to detoxify furfural compared to vanillin, and is in agreement with the results of Palmqvist et al.^6^, who observed that phenolic compounds found in lignocellulosic hydrolysates caused less inhibition than furfural^5,9^. The presence of vanillin and furfural simultaneously caused a faster and longer depolarization as the effects of both inhibitors work synergistically (Fig. 5d).

**Figure 8:**
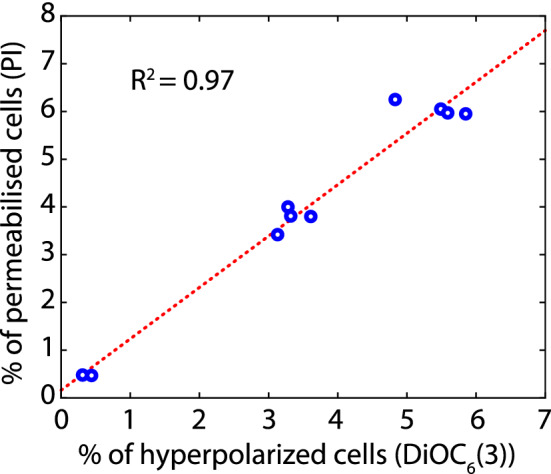
Correlation between % of hyperpolarized and % of permeabilized cells in experiment 8. The hyperpolarized cells were measured using DiOC_6_(3) and the permeabilised cells measured using PI. This figure was produced using Python^70^.

The immediate depolarization of the cell membrane caused by acetic acid (Fig. 5e–h) can be directly related to its mechanism of inhibition. Acetic acid penetrates the cell membrane via passive diffusion through the cell membrane, or facilitated diffusion using transmembrane transporters such as the aquaglyceroporin Fps1^59–61^. In all cases, the non-ionized form of acetic acid is preferred^62^, linking the diffusion rate to the external pH and the pKa of acetic acid. Upon entrance, the close-to-neutral cytosolic pH (between 6.5 and 7.5^63,64^) promotes the immediate dissociation of acetic acid, resulting in the acidification of the cytosol and in the subsequent loss of membrane potential. Narayanan et al.^31^ showed that yeast cells with a lowered cytosolic pH had a higher tolerance towards acetic acid because it reduces the fraction of dissociated acetic acid. This fast depolarisation of the membrane contrasts with the slower effects caused by vanillin or furfural due to the biochemical nature of the inhibition (Fig. 5a–d). To keep internal pH homeostasis, the protons are actively exported using an ATP dependent transporter^26,62,64^. As energy is used to balance the internal pH, the cell growth is interrupted^6^ and other cell properties such as the cell turgor, the amino acids pool or the mitochondrial integrity are also affected^32,61,62,65^. The inhibitory potential of acetic acid is therefore a balance between the inflow of acetic acid and the detoxification capabilities of the cells. When acetic acid was present together with vanillin, furfural or both, a small population of apparently hyperpolarized cells appeared at the end of the fermentation. A clear correlation between the percentage of hyperpolarised cells measured with (DiOC_6_(3)) and permeabilised cells measured with PI was observed within the same sample (Fig. 8), indicating that the hyperpolarized cells corresponded to dead cells. Moreover, Freitas et al.^32,33^, who also found this population during the stationary phase in cell cultures of *Saccharomyces carlsbergensis* grown with acetic or formic acid, showed that this hyperpolarized population corresponded to the fraction of cells with permeable membranes. However, it was not possible to know whether the apparent hyperpolarization is due to a real potential difference through the membrane or it is an artefact from unspecific staining of dead cells with permeabilised membranes.

The porosity of the cell membrane is commonly used to indirectly assess the viability of a cell culture, as stressed or damaged cells have pores in their membranes. However, several studies have shown that the degree of occurrence of pores depends on the stress factors and does not always relate to cell viability^22,53^. When assessed with PI, different degrees of fluorescence are found between non-stressed and non-viable cell cultures^53^. In previous studies examining the effect of lignocellulosic inhibitors on *S. cerevisiae*^31^ or the effects of acetic and formic acids on *S. carlsbergensis*^32,33^, PI was used as a binary indicator of cell viability, however, in this study, PI was used to assess the degree of damage of the cell membrane of to provide a more complete understanding of the physiological conditions of the cell membrane. This was found in the experiments 1–4, where the PI fluorescence increased during the stationary phase (Fig. 6a–d) indicating an increase in membrane permeability. The higher porosity was arguably induced by the accumulated ethanol during the fermentation as shown in previous studies^7,19,23,25^. In spite of the higher membrane porosity, the other physiological features (membrane potential (Fig. 5a–d) and intracellular ROS (Fig. 7a-d)) remained unaltered, suggesting that *S. cerevisiae* was able to maintain internal homeostasis despite having alterations in the membrane. This result demonstrates the high ethanol tolerance (able to tolerate ethanol concentrations of 18% v/v) of the industrial strain Ethanol Red® used throughout the experiments 1–8. In the presence of acetic acid (Fig. 6e–h), the membrane permeability barely changed during the fermentation and ethanol did not induce a higher porosity in the membrane. This suggests an acquired ethanol tolerance caused by an adaptive response to acetic acid. Lindberg et al.^14^ and Lindahl et al.^15^ studied the lipidomic profile of *S. cerevisiae,* showing that acetic acid triggers a modification in the membrane composition promoting the synthesis of complex sphingolipids^14,15^. In a genome-wide identification of the genes involved in acetic acid resistance, Mira et al.^65^ found that several genes involved in the synthesis of membrane structural lipids such as ergosterol, phospholipids and sphingolipids were overexpressed in the presence of acetic acid^65^. An increased fraction of ergosterol in the cell membrane has also been previously related to ethanol tolerance^12,13^, as it confers a reduced fluidity and a higher density of the cell membrane^20^. This indicates that the adaptive responses to acetic acid and to ethanol have mechanisms in common, explaining the reduced membrane permeability caused by ethanol in the experiments where acetic acid was present (Fig. 6e–h). Previous studies have shown an increased toxicity of acetic acid in the presence of ethanol^62^. This is due to an increased diffusion of acetic acid through the cell membrane due to the higher membrane porosity induced by the presence of ethanol. However, in the present study, this was not observed because acetic acid was detoxified before a high ethanol titer was reached (Fig. 2e–h). Endo et al.^16,17^ have shown that vanillin also increases the fraction of ergosterol in the membrane. An acquired ethanol tolerance as a consequence of the adaptation to vanillin would therefore be expected. However, this was not observed, most probably because the concentration of vanillin used in this study was 5 times lower than that used by Endo et al. During the experiments containing acetic acid together with vanillin, furfural or both, a second population of cells with high membrane permeability appeared (Fig. 6e–h). This population most probably corresponded to highly stressed cells due to the combined effects of the different inhibitors.

Analyzing the intracellular ROS compounds is of interest as prolonged accumulation causes DNA and protein damage and eventually programmed cell death, thereby reducing the viability of the cell culture^36^*.* All inhibitors increased the intracellular concentration of ROS compounds, indicating that they exert oxidative damage in the cells (Fig. 7a–h). The oxidative stress induced by phenolic compounds, furfural, or acetic acid has also been previously reported in the literature^26,56,66^. Wu et al.^18^ and Trinh et al.^55^, have also shown that furfural, 5-HMF, and vanillin induce the overexpression of genes related to ROS detoxification such as the *YAP1* gene in *S. cerevisiae*. The accumulation of the intracellular ROS scavenger trehalose has also been reported as a response to acid stress^45^. Despite all the experimental evidence showing that vanillin, furfural, and acetic acid induce oxidative stress, the specific mechanisms inducing the accumulation of ROS remain unclear to the best of our knowledge^56,67^. The increased accumulation of ROS correlated with a lower production of glycerol during the lag-phases (Figs. 2 and 7). This correlation probably reflects the dependence of the of the enzymatic system involved in the ROS detoxification with the redox state of the cells, as previously described by Herrero et al.^68^. However, more experiments are needed to elucidate the specific mechanisms correlating the redox state of the cell and ROS detoxification. Among the different inhibitors, acetic acid resulted in a higher accumulation of intracellular ROS, suggesting that acetic acid exhibited a higher toxicity than vanillin or furfural (Fig. 7a–h). Similar to the membrane potential, the intracellular accumulation of ROS did not increase when the membrane porosity increased towards the end of the experiments 1–4 (without acetic acid) (Fig. 6a–d). This shows that although the ethanol concentration in the media is high enough to increase the porosity of the membrane, it does not induce oxidative stress inside the cells, demonstrating the ethanol resistance of the strain used in this study.

The different inhibitors commonly found in lignocellulosic feedstocks cause physiological changes in *S. cerevisiae* that trigger a complex systemic response to keep the internal homeostasis and increase the tolerance towards the toxic compounds. Previous studies have found that the transcriptome^17^, proteome^10^, lipidome^14^, and metabolome^11^ of *S. cerevisiae* change as a response to the toxicity of the inhibitors. In this work, the changes of three physiological features (membrane potential, permeability, and intracellular ROS) of *S. cerevisiae* during different batch fermentations were systematically analyzed, providing a detailed picture of the response of *S. cerevisiae* to the different inhibitors. Moreover, the physiological changes analyzed with flow cytometry were related to the specific mechanisms of inhibition and adaptation previously described in the literature, giving a holistic understanding of the response of *S. cerevisiae* to the different inhibitors. The possibility to assess the physiological conditions of the cell culture and the ‘real-time’ nature of the data acquired with flow cytometry make the analysis of the membrane potential, permeability, and intracellular ROS concentration not only attractive from a research perspective in the development of improved strains, but also as an applied method to assist the development of new cell culture propagation strategies. In the last years, there has been an increased interest in studying the effects of the pre-adaptation of *S. cerevisiae* during the cell culture propagation to achieve a higher tolerance towards the inhibitors during the fermentation^69^. By assessing the physiological conditions of the cell culture it is possible to optimize the propagation strategies to achieve a pre-culture with a higher inhibitor tolerance, reducing the lag phase during the fermentation and increasing the process productivity.

## Conclusions

In order to improve the productivity of cellulose-to-ethanol fermentation processes it is necessary to develop yeast strains able to tolerate the inhibitors present in lignocellulosic biomass. Understanding how *Saccharomyces cerevisiae* responds to the different toxic compounds is fundamental to design improved strains. Using flow cytometry to examine changes in the physiology of the cell membrane of *S. cerevisiae,* such as the membrane potential or membrane permeability, reflected on the mechanisms of inhibition and provided a good indication of the status of the cell culture. This information is valuable in the design of new propagation strategies or could be used as to assess the performance of different candidate strains during the development of improved yeast strains.

## Materials and methods

### Cell culture and cultivation media

One milliliter glycerol stock of *Saccharomyces cerevisiae* Ethanol Red^®^ (Lesaffre Advanced Fermentations) was grown in 250 mL shake flasks with 100 mL of Yeast Peptone Dextrose medium (YPD), containing 10 g/L of yeast extract, 20 g/L of peptone and 20 g/L of glucose, for 15 h at 30 °C and 180 rpm, was diluted 1000 times, plated in a solid YPD-agar plate and incubated at 37 °C for 36 h prior to storage at 4 °C. The pre-culture was prepared by adding one single colony to 250 mL shake flasks with 100 mL of YPD medium, and was grown for 15 h at 37 °C and 180 rpm. Eight cultivations following a replicated 2^3^ full factorial experimental design were performed in 250 mL shake flasks containing 100 mL of fermentation media. The same batch of YPD medium was used throughout the experiments 1–8 to avoid the effects of variability in the YPD composition. The cultivation media were prepared independently, had the same YPD concentration, and the specified concentrations of inhibitors according to the experimental design (Table 1). The concentrations of each inhibitor were chosen to represent common concentrations present in wheat straw hydrolysate^42^. Each shake flask was inoculated with 5 mL of pre-culture to reach a final cell density OD_600_ of 0.75. The cultivations were performed at 37 °C, 180 rpm and at an initial pH of 5.5, lasted between 12 and 24 h and were stopped 2 h after the end of the exponential growth phase. Replicates of each cultivation were done from independently grown pre-cultures.

### Measurements of biomass and extracellular metabolites

One milliliter of the cultivation broth was taken hourly to measure the optical density at 600 nm (Shimadzu UV-1800, Japan). The growth rate of each experiment was calculated from the slope of the linear section of the logarithm of the optical density. The lag-phase was calculated as the time where the logarithm of the optical density becomes linear. Every 2 h, 1 mL of fermentation media was taken, filtrated through a 0.20 µm cellulose acetate filter (Labsolute USA, 7699822) and stored at − 20 °C for HPLC analysis. The HPLC analysis was performed using an Ultimate3000 HPLC system. Glucose, ethanol, acetic acid, glycerol, pyruvate, vanillin and furfural were separated using an Aminex HPX-87 H column (BIORAD, USA) at 40 °C with 5 mM H_2_SO_4_ as eluent and a flow rate of 0.6 mL/min. Prior to injection, 950 µL of the samples were diluted with 50 µL of 5 M H_2_SO_4_. The total run time of the method was 120 min. The refractive index (at 50 °C) was used to detect glucose, ethanol, acetic acid, glycerol and furfural. Vanillin and pyruvate were detected by UV (205 nm) absorbance. The chromatogram of each sample was collected and analysed using Chromeleon 7.2. The retention time of glucose, ethanol, acetic acid, glycerol, pyruvate, vanillin and furfural were identified using standard solutions, and their concentration was quantified using a calibration set for each compound, that linearly correlates the area under each peak with the concentration of the corresponding compound. A glucose standard was run every ten samples to allow detection of potential occurrence of drift in the instrument.

### Analysis with flow cytometer

#### Experimental methods and sample preparation

Every 2 h, a 3 mL sample was withdrawn from the fermentation media for analysis of the cells with flow cytometry. Prior to analysis, the 3 mL sample was split into three sub-samples of 1 mL which were diluted to reach an OD_600_ of 0.2. One milliliter of each diluted sub-sample was stained with either dihydrorhodamine 123 (DHR123), 3,3′-dihexyloxacarbocyanine iodide (DiOC_6_(3)) or propidium iodide (PI). The stock solution of DHR123 was prepared by dissolving 10 mg of DHR123 (Thermo Fisher Scientific, D632) in dimethyl sulfoxide (DMSO > 99.7%, Merck, 34869) to reach a concentration of 1 mM. The final volume was distributed in 1 mL Eppendorf tubes and stored at − 20 °C until used. The staining with DHR123 was done first by adding 40 µL of the stock solution to 1 mL of cell suspension diluted with demineralized water to reach a final OD_600_ of 0.2 and then by incubating the samples in the dark at 37 °C for 10 min prior to analysis. The stock solutions of DiOC_6_(3) were prepared by dissolving 100 mg of DiOC_6_(3)) (Thermo Fisher Scientific, D273) in DMSO to a concentration of 17.5 mM. They were distributed in 1 mL Eppendorf tubes and stored at − 20 °C until use. Prior to staining, the DiOC_6_(3) stock solution was diluted 1000 times with deionized water. A volume of 23 µL of the diluted solution was added to 1 mL of cell suspension diluted with demineralized water to reach an OD_600_ of 0.2. The samples were then incubated in the dark at 37 °C for 10 min. After the incubation, the samples were centrifuged at 1800 rpm for 5 min. Whereas he supernatant was discarded, the cells were re-suspended in 1 mL of demineralized water and immediately analyzed. One milliliter of a 1 mg/mL propidium iodide solution in water (Thermo Fisher Scientific, P3566) was directly added to 1 mL of suspended cells (diluted with demineralized water to a final OD_600_ of 0.2). The final PI solution was incubated at 37 °C for 10 min. The staining procedures for DiOC_6_(3) and DHR123 were based on the protocol described by Freitas et al.^32^, and validated using two cell cultures of *S. cerevisiae* Ethanol Red®, a non-stressed cell culture incubated in YPD at 37 °C for 3 h and a stressed cell culture incubated in YPD with 7.5 g/L of acetic acid at 37 °C for 3 h. The staining procedure for PI was developed following the product recommendations by Thermo Fisher Scientific, and validated using a non-stressed cell culture of *S. cerevisiae* Ethanol Red^®^ grown in regular YPD at 37 °C for 3 h, and a stressed cell culture incubated in YPD with 70% v/v of ethanol at 37 °C for 1 h. Artificial samples with different ratios of stressed and non-stressed cells were created, stained with the corresponding procedure for each stain and subsequently analysed with flow cytometry. The resulting cytograms of each artificial sample showed the corresponding ratio between stressed and non-stressed cells. Finally, negative controls for each staining procedure were made, including the samples from the non-stressed and stressed cell cultures where the stains were substituted with demineralized water.

#### Data acquisition

The stained samples were analyzed by a BD FACS Jazz Cell Sorter (BD Biosciences, USA) equipped with a 488 nm argon ion laser. The green fluorescence emitted by DHR123 or DiOC_6_(3) stained samples was collected with a band-pass filter of (530 ± 40) nm, whereas samples stained with PI (red fluorescence) were collected with a band-pass filter of (629 ± 40) nm. 8 peaks Rainbow Calibration Samples (BD, 559123) were used at the beginning of each experiment to calibrate the instrument, and were run every 4.5 h for quality control. For each analyzed sample, 10,000 events were recorded at an event rate between 1800 and 2000 events per s. The number of events per sample was determined based on the number of events required to keep the population properties (mean and standard deviation) constant, which happened after collecting 5000 events. Together with the fluorescence, the forward (FSC) and side scatter (SSC) were also collected as they provide valuable information regarding physical properties of the cell. The data were recorded using the BD FACS Software (BD Biosciences, USA).

#### Data analysis and visualization

**Figure 9:**
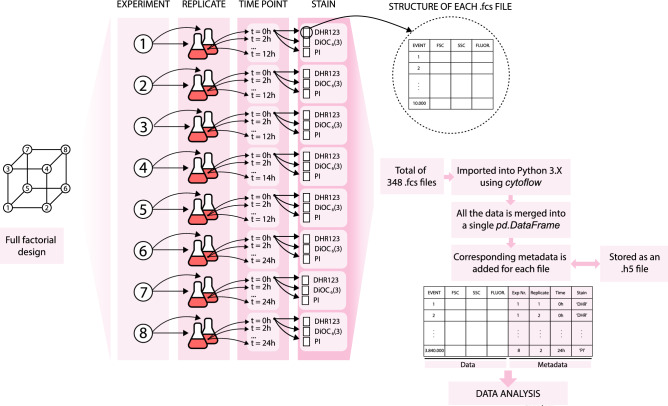
Summary of the experimental design and visualization of the data set structure.

The data obtained from optical density and HPLC measurements were directly imported and analyzed in Python 3.5^70^. The data from the flow cytometry was imported and analysed in Python 3.5 using the *cytoflow* library. Data from flow cytometry consisted of 348 .fcs files containing 10.000 events each. Each file corresponded to a different experiment, time point, stain and replicate. All 348 .fcs files were merged into a single large data-frame, which included the metadata of each file (experiment, time point, stain, replicate and day of the experiment). Importing the 348 .fcs files individually is very time-consuming, for this reason, once imported, the data-frame was saved as an .h5 file, which can be loaded much faster than the 348 .fcs files. Statistical and exploratory analysis were conducted using the *scipy, numpy and pandas* Python libraries. All data was scaled to the mean value of the population from experiment 1 at time 0 h, which was considered to be a healthy cell culture. Hence, all data is expressed relative to that physiological state. Data visualization (2D density plots) was done using the *seaborn* library. Sequential 1D density plots (ridge plots) were done in R 3.5.1^71^ (libraries *ggplot2* and *ggridges*) after exporting the data from Python as a .csv file. A summary of the experimental design and how it was translated into the data-frame is depicted in Fig. 9.

To validate the experimental protocol and to evaluate the quality of the data obtained with the flow cytometer, the cell distributions from each replicate were compared by analysing the weighted error distribution of all pairs of replicates. That is, for a given sample (experiment, time point, and stain), a cell distribution was measured for each of the two replicates, where each of them was approximated to a normal distribution with mean µ and standard deviation σ (${X}_{1}\sim N\left({\mu }_{1},{\sigma }_{1}\right)$ and ${X}_{2}\sim N\left({\mu }_{2},{\sigma }_{2}\right)$). Ideally, ${X}_{1}$ and ${X}_{2}$ should belong to the same distribution, but due to experimental errors and to natural biological variations, they do not. Therefore, in order to assess how much the replicates differ from each other, the distance between their means was evaluated by using the equation (1) below:

1 $$ error = \frac{{\left| {\mu _{1}  - \overline{\mu } } \right|}}{{\overline{\mu } }} $$

where $\overline{\mu }  = \left( {\mu _{1}  + \mu _{2} } \right)/2$. This error was calculated for all the 124 replicated samples, and the distribution of errors was used to analyze the quality of the collected data.

## Supplementary information

Supplementary figures.

## Data Availability

The datasets generated during and/or analysed during the current study are available in the FlowRepository, http://flowrepository.org/id/RvFr2O6sLRPP2nUJwNd5az7rBm2EipS6agdp7F3Wxk9trkhxEoPBnWKsB7MpnTLU.
