# Supplementary material for: Analysis of the response of the cell membrane of Saccharomyces cerevisiae during the detoxification of common lignocellulosic inhibitors
Source: Sci Rep. 2021 Mar 25;11:6853. doi: 10.1038/s41598-021-86135-z (PMC7994549; doi:10.1038/s41598-021-86135-z)
Supplement: Supplementary file 1 — Supplementary figures. [file 41598_2021_86135_MOESM1_ESM.pdf]

Title: Analysis of the response of the cell membrane of *Saccharomyces cerevisiae* during the detoxification of common lignocellulosic inhibitors

Pau Cabaneros López[1], Chuantao Peng[2], Nils Arneborg[2], Helena Junicke[1], Krist V. Gernaey[1,*].

[1]*Process and Systems Engineering Center (PROSYS), Department of Chemical and Biochemical Engineering, Technical University of Denmark (DTU), Building 228A, 2800 Lyngby, Denmark.*

[2]*Department of Food Science, University of Copenhagen (KU), Rolighedsvej 26, 1958 Frederiksberg C, Denmark.*

Corresponding author: Krist V. Gernaey, kvg@kt.dtu.dk

Journal: Scientific Reports

Keywords: Flow cytometry, cellulosic-ethanol, single-cell analysis

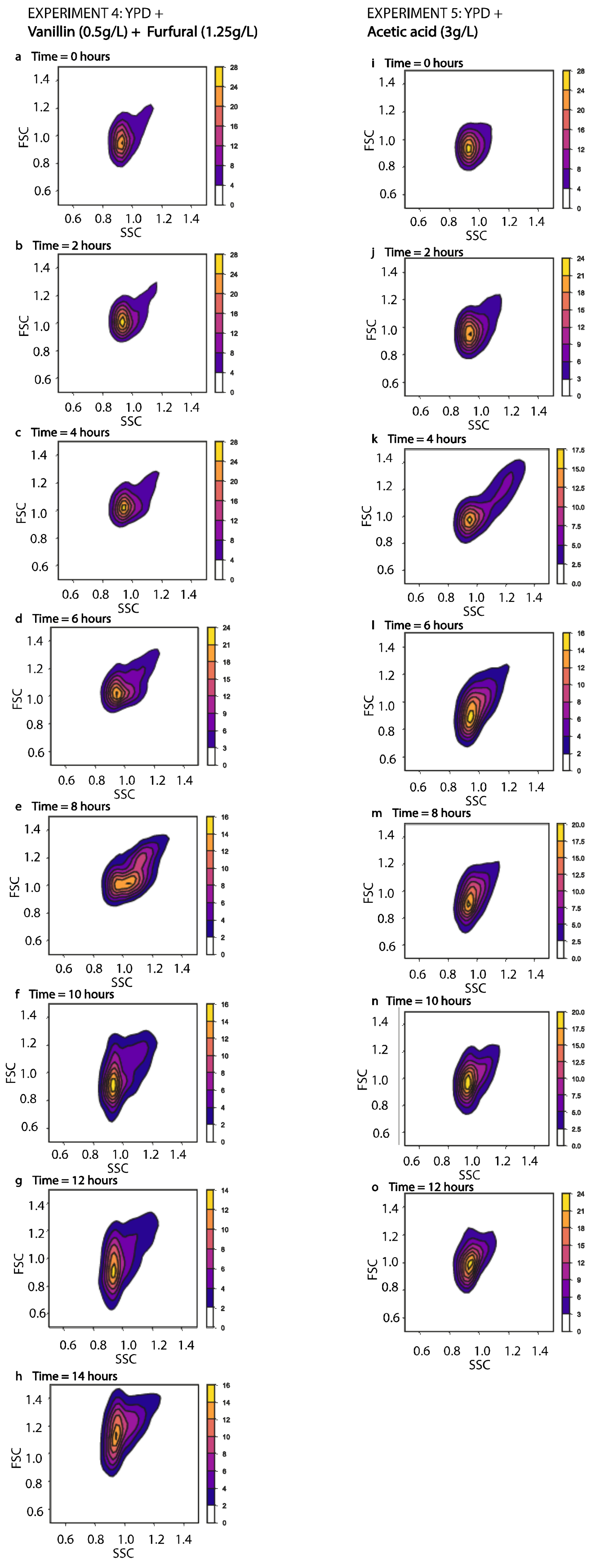
EXPERIMENT 4: YPD +
Vanillin (0.5g/L) + Furfural (1.25g/L)
a Time = 0 hours
b Time = 2 hours
c Time = 4 hours
d Time = 6 hours
e Time = 8 hours
f Time = 10 hours
g Time = 12 hours
h Time = 14 hours
EXPERIMENT 5: YPD +
Acetic acid (3g/L)
i Time = 0 hours
j Time = 2 hours
k Time = 4 hours
l Time = 6 hours
m Time = 8 hours
n Time = 10 hours
o Time = 12 hours
FSC
SSC
0.6
0.8
1.0
1.2
1.4

Figure SM1. Two-dimensional density plots showing the evolution of the forward and side scatter. The density plots correspond to experiment 4 (SM1.a-g) and experiment 5 (SM1.h-o). Notice that the FSC and SSC are scaled by the mean FSC and SSC of experiment 1 at time 0 hours. Hence, all measured changes in the population of cells are given in relation to that state. The colour gradient shows the density of the distribution in the populations, with yellow corresponding to the highest density, and blue corresponding to the lowest one.

EXPERIMENT 6: YPD +
Vanillin (0.5g/L) + Acetic acid (3g/L)

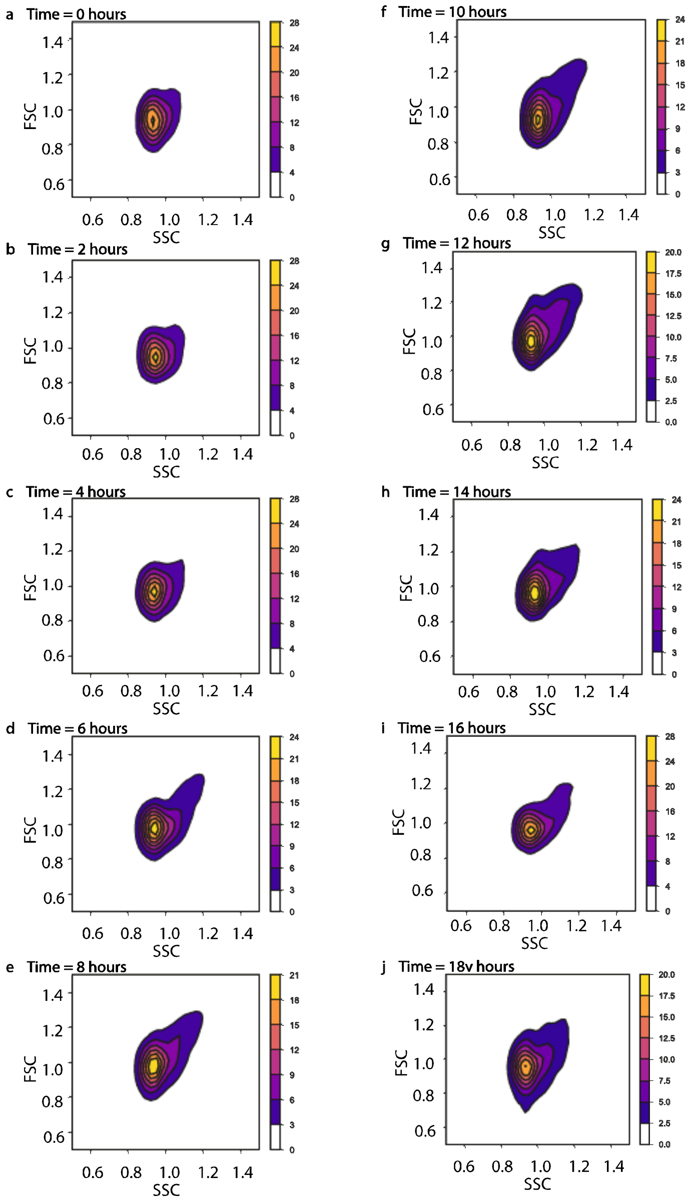

Figure SM2. Two-dimensional density plots showing the evolution of the forward and side scatter. The density plots correspond to experiment 6 (SM2.a-j). Notice that the FSC and SSC are scaled by the mean FSC and SSC of experiment 1 at time 0 hours. Hence, all measured changes in the population of cells are given in relation to that state. The colour gradient shows the density of the distribution in the populations, with yellow corresponding to the highest density, and blue corresponding to the lowest one.

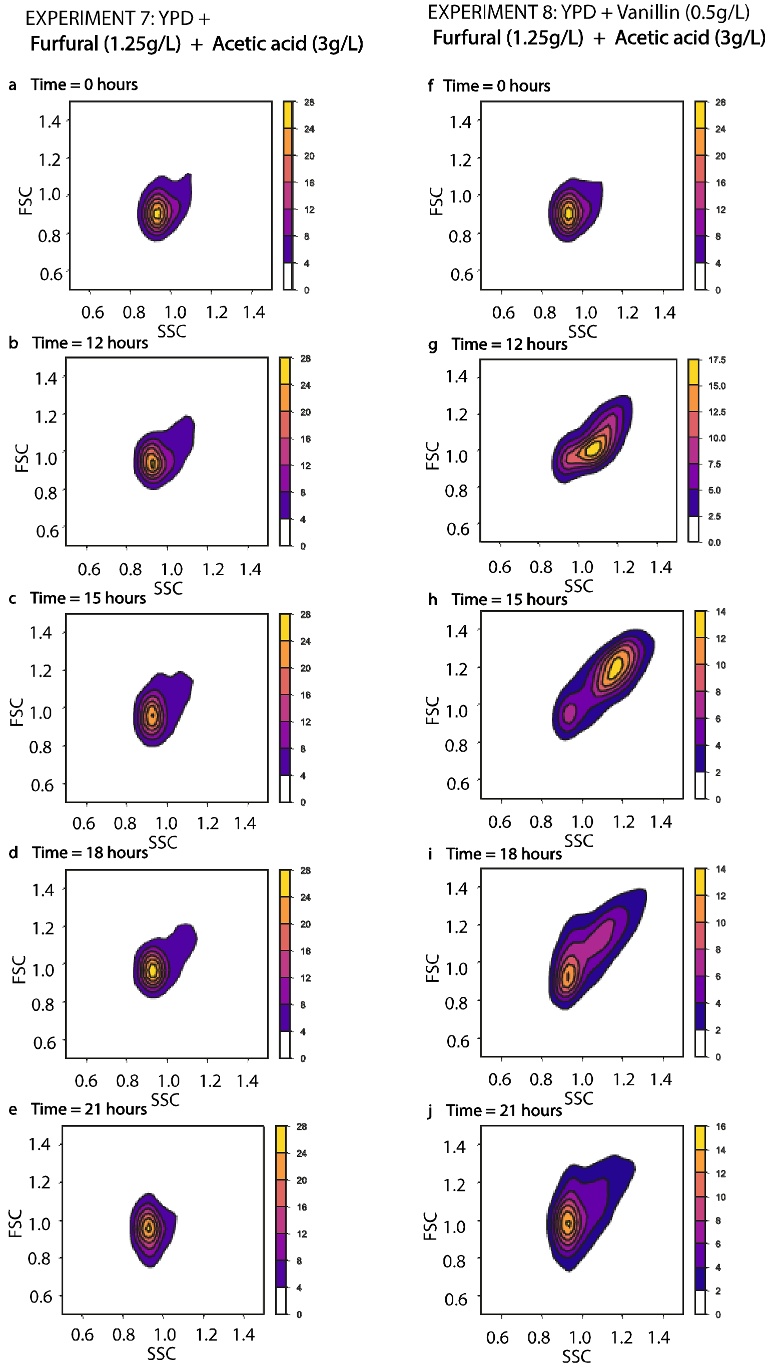

Figure SM3. Two-dimensional density plots showing the evolution of the forward and side scatter. The density plots correspond to experiment 7 (SM3.a-e) and experiment 8 (SM3.f-j). Notice that the FSC and SSC are scaled by the mean FSC and SSC of experiment 1 at time 0 hours. Hence, all measured changes in the population of cells are given in relation to that state. The colour gradient shows the density of the distribution in the populations, with yellow corresponding to the highest density, and blue corresponding to the lowest one.
